# The Changing Epidemiology of Sudden Infant Death Syndrome: A 15‐Year Overview Comparing Italian and European Data

**DOI:** 10.1002/hsr2.70599

**Published:** 2025-03-23

**Authors:** Matteo Manfredini, Serafina Perrone, Alessia Ardenghi, Anna Maria Lavezzi, Virginia Beretta, Elena Scarpa, Sabrina Moretti, Susanna Maria Roberta Esposito, Laura Filonzi, Francesco Nonnis Marzano

**Affiliations:** ^1^ Department of Chemistry, Life Sciences and Environmental Sustainability University of Parma Parma Italy; ^2^ Department of Medicine and Surgery, Neonatology Unit University Hospital of Parma Parma Italy; ^3^ Lino Rossi Research Center University of Milan Milan Italy; ^4^ Department of Medicine and Surgery, Pediatric Clinic University Hospital of Parma Parma Italy

**Keywords:** back to sleep campaign, epidemiological data, perinatal medicine, SIDS incidence, Sudden Infant Death Syndrome, Sudden Unexpected Infant Death (SUID)

## Abstract

**Background and Aims:**

Sudden Infant Death Syndrome (SIDS) represents a prominent cause of infant death in many countries. Epidemiological data has been variable over time because the related International Classification of Diseases (ICD) code is not consistent throughout countries and has changed over the years. The prevalence of SIDS is unclear, with estimates that do not reflect the number of patients who actually died from SIDS. This paper aims to assess the trend of SIDS in Italy and Europe during 2011–2018, and factors contributing to epidemiological data. Data for Italy were also integrated with an individual‐level analysis over the period 2003–2018.

**Methods:**

A two‐pronged analysis was performed starting from the Italian National Institute of Statistics and experimental data. The individual characteristics of SIDS infants were detailed in association with biomedical, socioeconomic, and cultural variables.

**Results:**

Total infant mortality has been continuously declining in Italy, from 4.15‰ in 2003 to 3.05‰ in 2018 (−26.5%) with rates significantly lower than the European average in the same period (mean Italy 3.05‰ vs. mean Europe 4.11‰). Considering only SIDS, the 28 European countries show an average value of 0.15/1000 deaths/births (2011–2018), with a decreasing temporal trend. Italy displays an average rate 75% lower (0.04/1000 births). The seasonality of the syndrome highlights a prevalence during cold months (60.7%) and no evidence of a significant effect of mother's age at birth was found. The mean age at death is prevalent in the postneonatal period. No statistically significant effects on Italian SIDS mortality have been found regarding economical, educational, and cultural aspects related to the care of infants.

**Conclusion:**

The data suggest a likely effect of different ways of classification of SIDS‐related deaths, although a different approach to the prevention campaigns could be responsible for data variability among countries. Results also suggest an urgent need to get insight into previously unexplored aspects, such as neuroanatomical, genetic, metabolic, and proteomic aspects, focusing especially on high‐risk groups to further clarify the etiopathogenesis of this syndrome.

## 1 Introduction

The Sudden Infant Death Syndrome (SIDS) is classified as a death cause having an independent nosology category (code R95) in the Italian system of death classification. The syndrome is inserted in the more generic SID (Sudden Infant Death) category whose classification refers to a wider spectrum of causes mentioned with codes in the range R00‐R99. At the multinational level, SIDS, first defined in 1969 [1], did not have a unique International Classification of Diseases (ICD) code assigned until the ICD 9th revision. At that time SIDS was introduced worldwide as 798.0. The code was subsequently changed in R95 in ICD‐10 [2]. However, the coding system appears not to be consistent throughout countries as additional codes are often randomly assigned [3]. Similarly, epidemiological data has been variable worldwide during the years [4, 5]. These two topics are strictly intertwined as infant mortality rates for SIDS tend to vary according to the proposed cause of death codes [6].

Historically, SIDS is referred to as a sudden death within the first year of age, occurring without any cause or valid scientific explanation even after an autoptic exam [7]. Although an autonomic nervous system dysfunction in the control of cardiocirculatory and/or respiratory activity has been evidenced [8, 9], the syndrome etiopathogenesis is still obscure and several risk factors have been hypothesized [10, 11, 12, 13] within the so‐called “Triple Risk Model” (according to [14], SIDS occurs at the intersection of three specific situations: (1) a vulnerable infant, (2) a critical developmental period in homeostatic control, and (3) an exogenous stressor).

SIDS mortality has decreased by over 50% for most countries after the “back to sleep” campaigns introduction that has had a great impact on reduction of death rates [15]. On the other hand, perinatal care has experienced several other improvements, so it is difficult to focus the SIDS incidence drop to only the supine sleep practice [15]. Furthermore, major attention dedicated to additional possible causes may represent another explanation for the decline in SIDS rates [16]. Despite continuous public health efforts to improve sleep conditions, with a special focus on the high‐risk groups (ethnicity, poor socioeconomic status, maternal smoking during pregnancy and postnatal periods, preterm birth, history of maternal addiction, bottle‐feeding, and subclinical infections), SIDS incidence continues to be high and represents a prominent cause of infant death in many countries [10, 17, 18]. In the United States, 8% of all infant deaths are still attributed to SIDS, only behind congenital malformations and chromosomal abnormalities (21%) and disorders related to prematurity and low birth weight (17%) [19]. In relation to all potential variables, a quantitative evaluation of the SIDS impact within an infant mortality comprehensive framework has always posed a challenge. Difficulties are represented by the diagnostic uncertainty in referring to true SIDS as the primary cause of child death. On the other hand, the official statistics have been explicitly reporting SIDS as a codified cause of death for only a few years, 2003 for the Italian National Institute of Statistics (ISTAT), and 2011 for the European statistical office (EUROSTAT).

Italian national institutions provide data that allow a quantitative assessment of the prevalence and impact of the disease. Nowadays, the official incidence is about 1 per 1000 live births [10]. However, aggregate data provides a limited perspective to investigate the determinants of SIDS. Individual‐level data are much more indicated for that scope. In Italy, however, the collection of such data is often delegated to regional agencies, which are often uncoordinated with each other and thus unable to provide a representative picture of the phenomenon at the national level.

In consideration of the above aspects, this paper aims to assess the trend of SIDS in Italy and Europe during 2011–2018, and to define the epidemiological characteristics of SIDS in Italy in the period 2003–2018, using both aggregate and individual‐level data.

## 2 Materials and Methods

Aggregate data analyzed in this study are open‐access data publicly available from the Italian National Institute of Statistics (ISTAT) and the European Statistical Office (EUROSTAT). The individual data set is composed of 90 cases, comprehensive of anamnestic data (sex, maternal age, age at death, seasonality). In relation to their geographical distribution, 96.7% of cases were from northern Italy and 3.3% from central Italy. The individual‐level data set was kindly provided by Lino Rossi Research Center, which is the national reference center located at the University of Milan. Permission from the Ethics Committee was not required for this study as the Research Center is the national reference center for Sudden Unexplained Infant Death, in accordance with the Italian Law n. 31/2006 “Regulations for Diagnostic Post Mortem Investigation in Victims of SIDS and Unexpected Fetal Death”. This law decrees that all infants who die suddenly in Italian regions within the first year of age, as well as all fetuses who die without any apparent cause, must undergo, after obtaining informed parental consent, an in‐depth necroscopic examination, particularly of the autonomic nervous system. All individual cases were therefore authorized by the above cited law.

From a methodological point of view, the indicators of infant mortality used in the paper are the traditional infant mortality rates, calculated according to the following formulas:

$$\text{Overall Infant}=\frac{D_{0-365}^{t}}{N^{t}}\text{Neonatal}=\frac{D_{0-29}^{t}}{N^{t}}\text{Postneonatal}=\frac{D_{30-365}^{t}}{N^{t}-D_{0-29}^{t}}$$

where *N* ^t^ is the number of live births in year t (or period t), *D* ^t^ is the number of deaths in the same year (period) by age bracket: over the whole year (0–365 days), in the first month (0–29 days), or in months 1 to 12 (30–365 days). The rates are expressed in number of deaths per 1000 live births.

Rates for SIDS are based on the same formulas, with deaths by SIDS in place of total deaths in the numerator. SIDS rates are expressed in number of deaths by SIDS per 100,000 live births. Given the difficulties in arriving at a specific diagnostic classification of SIDS, it is often suggested to use the broader group of causes called SUID (Sudden Unexpected Infant Death) [20]. This group includes two further nosological categories, namely Unknown and Unspecified Causes of Death (code R99) and Accidental Suffocation and Strangulation in Bed (code W75). Unfortunately, ISTAT does not publish data for the group W75 due to the institute's adoption of a shortlist of causes of death that does not include publication of groups with very few cases. Alternatively, the official statistics, both at the national and European level, allow analyzing the entire R group of causes of death (symptoms, signs, abnormal findings, and ill‐defined causes, R00‐R99) to which SIDS belongs. Therefore, analyses on deaths from both SIDS and the R group have been carried out.

A linear regression model was estimated to determine the role of a set of socioeconomic variables on SIDS incidence in different European countries. The analysis covers the period 2011–2018 for reasons of data availability and robustness of official statistical indicators, and all the variables included in the model are therefore mean values over the same period. Among the variables analyzed, the average Gross Domestic Product per capita (GDP) was chosen as a proxy of economic welfare. Two indicators were included to capture parents' social and cultural background, often associated with the ways and habits of raising children: the percentage of foreign births and the percentage of women 20–54 years old with at least upper secondary education. These variables can be intended as proxies for women's lack of information about available maternal services and assistance, low response to campaigns promoting secure and correct behaviors to prevent sudden infant deaths, and delayed or inadequate obstetric care. These are the reasons why in a recent study on Italian infant mortality, foreign births were found to experience higher rates than the Italian ones [21].

Finally, the inclusion of the average country level of infant mortality aims at understanding whether SIDS mortality was somehow associated with the same factors affecting overall infant mortality. A quadratic element was added to test whether this relationship might change at different values of infant mortality. The coefficients reported in the table and discussed in the text represent the change in the SIDS mortality rate for each unitary change in the explanatory variables. A two‐tailed *t*‐test was used to test the hypothesis that each coefficient is different from 0. Moreover, an *F* test was used to check whether or not the estimated linear regression model provides a better fit than a model with no explanatory variables. Absence of significant multicollinearity among the explanatory variables was preliminarily ascertained using the *Variance Inflation Factor*.

The Fisher exact test was applied for the analysis of independence between two categorical variables, and two‐tailed unpaired *t*‐test was used for the analysis of the equality of two sample means. The *α* level of statistical significance is set to 0.05 for every test.

## 3 Results

### 3.1 Aggregate Data

**Figure 1:**
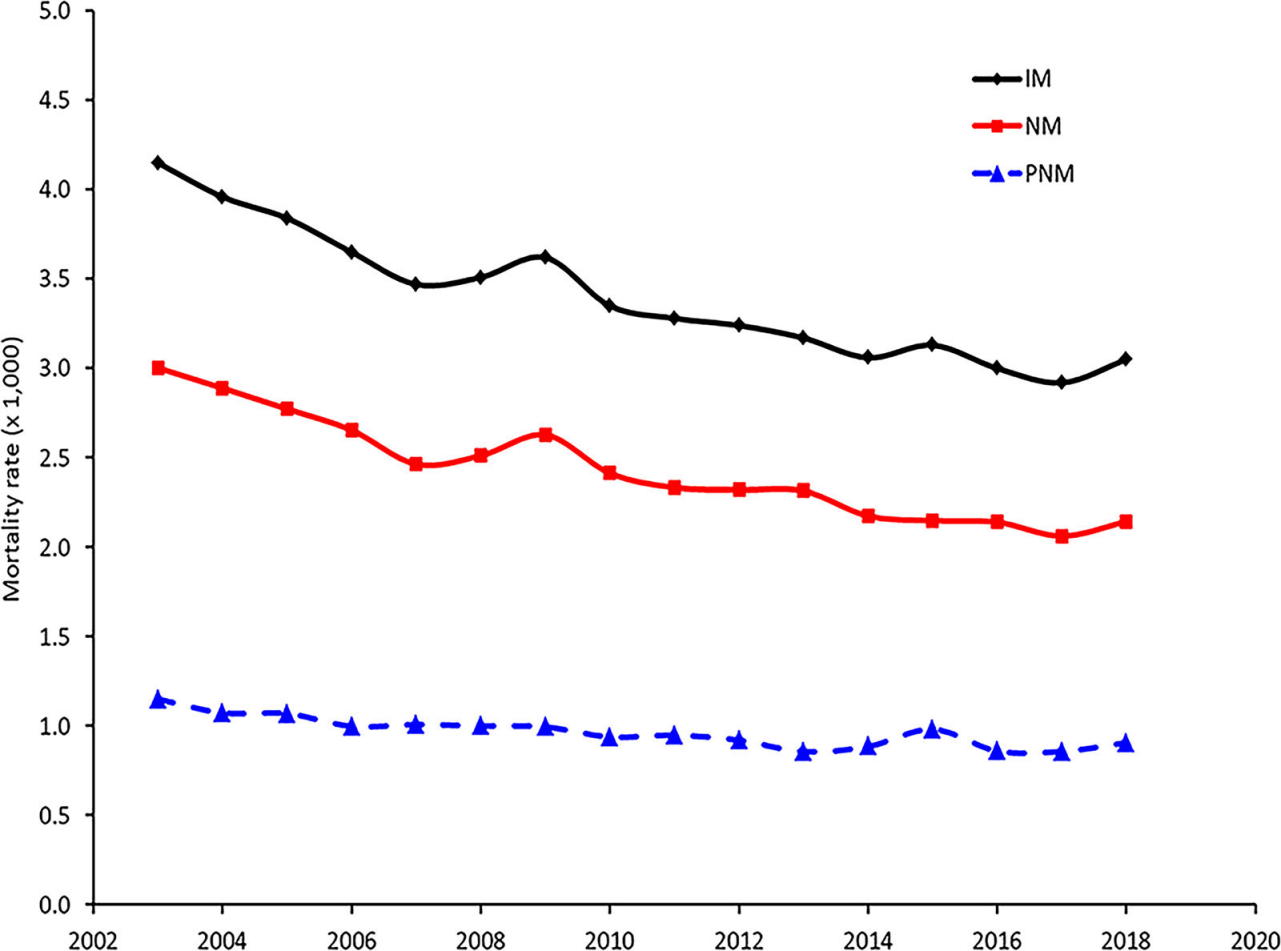
Infant, neonatal, and postneonatal mortality rates (× 1000), Italy 2003–2018. According to the ISTAT definition, data of infant mortality are referred to the territory of the event, thus concerning the population present in the national territory. These levels may slightly vary from those measured by territory of residence. IM = infant mortality; NM = neonatal mortality; PNM = postneonatal mortality.

Between 2003 and 2018, the Italian infant mortality rate (IM) dropped from 4.15‰ to 3.05‰. The postneonatal component (PNM, 1–11 months), with values steadily below 1‰ since 2009, had a major impact on such low levels than neonatal mortality (NM, 0 months), whose rates are always above 2‰. This latter, however, was characterized by a more marked downward trend, with reductions between 2003 and 2018 of 28.6% (Figure 1).

**Figure 2:**
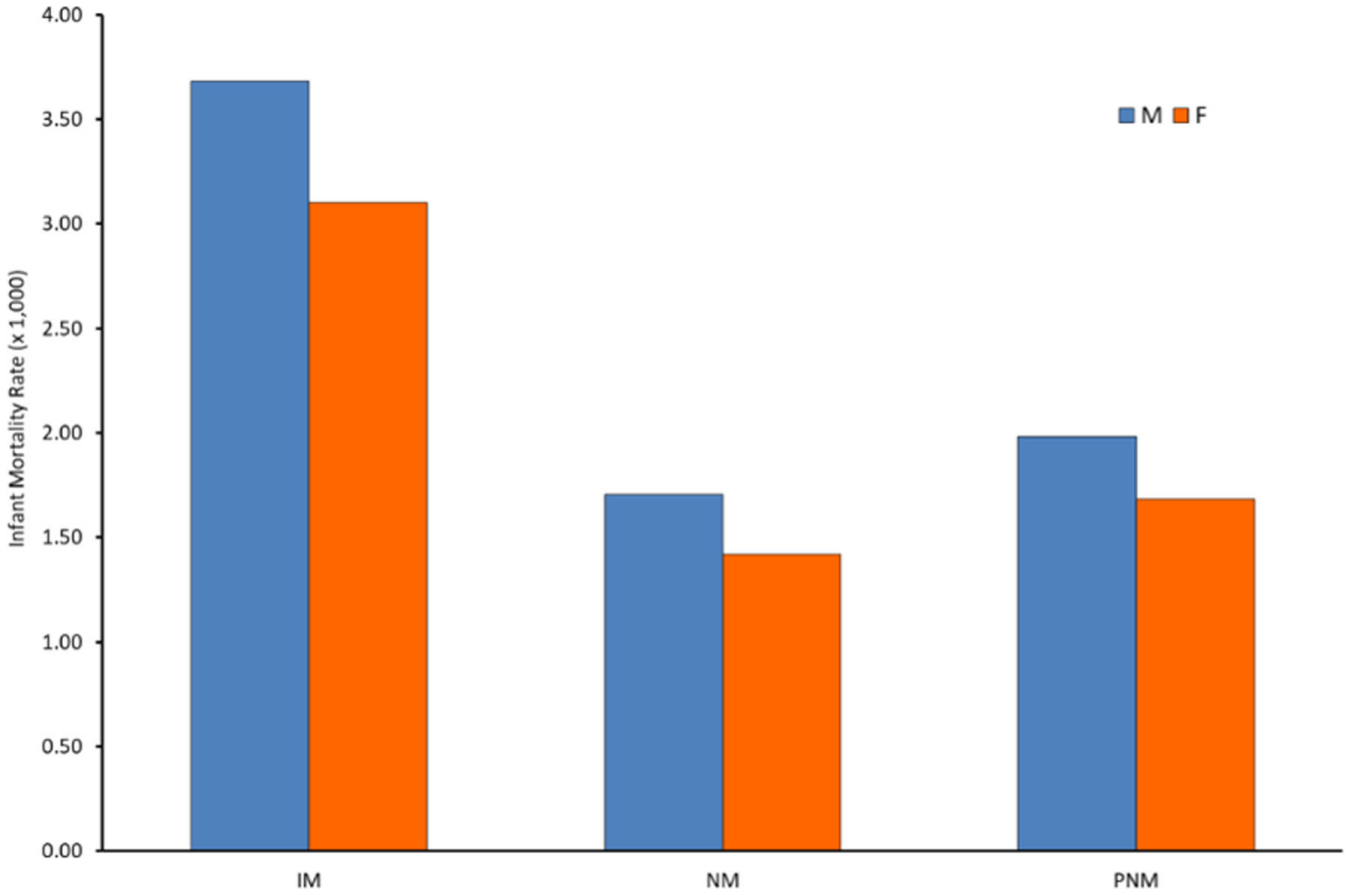
Infant, neonatal, and postneonatal mortality rates (× 1,000) by gender, Italy 2003–2018. IM = infant mortality; NM = neonatal mortality; PNM = postneonatal mortality.

Males have higher infant mortality than females [22], with an over‐mortality of about 19% between 2003 and 2018. This pattern also characterizes the neonatal and postneonatal period (Figure 2).

**Figure 3:**
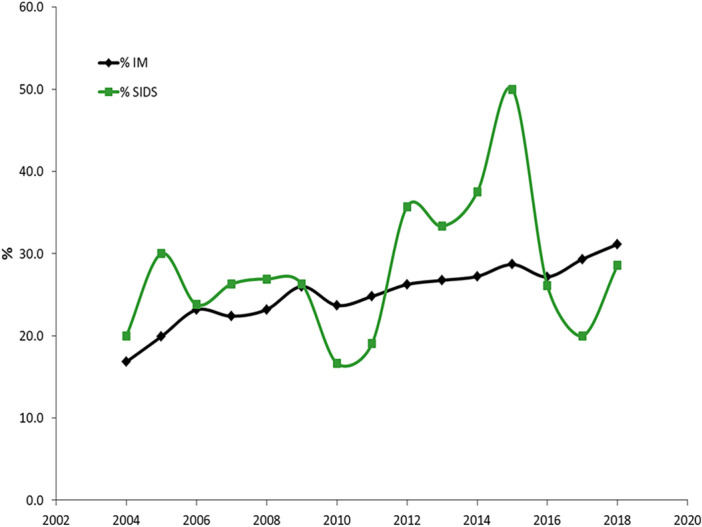
Percentage of foreign infant deaths out of total deaths within the first year of life. Italy, 2004–2018. IM = infant mortality.

The levels of Italian infant mortality remain very low (mean 3.05‰) with respect to the European value (4.11‰), despite the non‐marginal role of foreign infant deaths. According to ISTAT, their percentage on the total number of infant deaths in the period 2004–2018 shows an increasing trend, with an average of 25.1% (Figure 3).

As for SIDS, the Italian mortality rate in the first year of life is rather oscillating due to the lower number of cases involved. The average rate is 4.05 per 100,000 births over the period 2003–2018, fluctuating between 2.65 and 5.15 (Table 1). This means that about 21 infant deaths occurring each year in Italy are attributable to SIDS. It contributes therefore to only 1.2% of total deaths within the first year of life, with an irrelevant impact in the neonatal phase (0.4%), and a slightly greater one in the postneonatal period (3.3%).

**Table 1:** SIDS mortality rates in Italy (× 100,000 births) by year and age.

Year	< 1 year	< 1 month	1–11 months
2003	3.69	0.78	2.92
2004	4.93	1.28	3.65
2005	3.64	0.36	3.28
2006	4.13	1.62	2.52
2007	4.07	1.77	2.30
2008	5.10	0.70	4.39
2009	3.54	0.71	2.83
2010	4.32	1.26	3.06
2011	4.25	0.92	3.33
2012	2.65	0.19	2.46
2013	4.96	1.39	3.57
2014	3.86	0.00	3.86
2015	3.38	0.84	2.53
2016	5.15	0.86	4.29
2017	3.72	1.53	2.19
2018	3.41	0.45	2.96

As for differentials by gender, the mean sex ratio of infant mortality rates is 1.42, which implies a 42% higher mortality rate for males than females, a level not much different from that for the postneonatal period (1.51). In the neonatal period, the few cases of SIDS highlight some sort of gender equilibrium (0.972).

Finally, the foreign component of infant deaths had a slightly lower impact on SIDS than on overall infant mortality (32.6% and 34.9% on average, respectively), although this difference was not statistically significant (Fisher exact test, *p* = 0.41).

The analysis for the whole R group does not show any substantial difference from that for SIDS alone. The average death rate from causes belonging to that group is 0.124‰ in 2003–2018, 12 deaths per 100,000 births. Thus, the number of deaths from SIDS accounts for about one‐third of the total deaths in the R group (32.7%). In conclusion, the entire R group of causes of death remains at a rather low prevalence level, with limited impact on overall infant mortality.

At the European level, Italy shows a mean SIDS mortality rate in the period 2011–2018 that is 75% lower than the EU average, which is attested at continental level to about 15 deaths per 100,000 live births (Table 2). Interestingly, levels like those of Italy are experienced in some countries of the Balkan area, such as Romania, Bulgaria, Greece, and Serbia, as well as in some Northern European countries (Netherlands and Denmark). Conversely, the most populated countries of Europe—France, Germany, and the United Kingdom—show higher levels of SIDS mortality, with values ranging from about 17 per 100,000 to 25 per 100,000, thus 4–6 times higher than Italy.

**Table 2:** SIDS mortality rates in Europe, 2011–2018 (× 1000 births).

	2011	2012	2013	2014	2015	2016	2017	2018
EU‐28 countries	0.17	0.17	0.17	0.15	0.14	0.15	0.12	0.14
Belgium	0.24	0.24	0.25	0.21	0.17	0.19	0.03	0.09
Bulgaria	—	0.12	0.05	0.07	0.03	0.05	0.05	0.06
Czechia	0.17	0.09	0.15	0.24	0.18	0.10	0.24	0.16
Denmark	0.03	0.10	0.07	0.07	0.15	0.10	0.10	0.06
Germany	0.22	0.19	0.22	0.17	0.17	0.17	0.17	0.15
Ireland	0.25	0.28	0.29	0.25	0.21	0.19	0.26	0.21
Greece	0.02	0.01	0.04	0.02	0.01	0.04	0.06	0.08
Spain	0.13	0.11	0.11	0.12	0.11	0.11	0.12	0.10
France	0.24	0.25	0.23	0.20	0.21	0.20	0.18	0.24
Croatia	0.19	0.17	0.30	0.30	0.11	0.19	0.16	0.24
Italy	0.04	0.03	0.05	0.04	0.03	0.05	0.04	0.03
Latvia	0.64	0.65	0.34	0.41	0.45	0.32	0.34	0.41
Lithuania	0.20	0.10	0.10	0.26	0.19	0.13	0.14	0.04
Hungary	0.20	0.18	0.23	0.16	0.13	0.20	0.17	0.19
Netherlands	0.08	0.08	0.09	0.06	0.04	0.08	0.06	0.07
Austria	0.20	0.18	0.16	0.13	0.21	0.11	0.10	0.09
Poland	0.15	0.13	0.11	0.11	0.11	0.12	0.01	0.08
Romania	0.04	0.02	0.04	0.06	0.06	0.06	0.02	0.05
Slovenia	0.09	—	0.14	0.05	0.05	0.15	0.05	0.00
Slovakia	0.13	0.52	0.31	0.43	0.41	0.38	0.26	0.48
Finland	0.17	0.20	0.19	0.24	0.11	0.15	0.10	0.15
Sweden	0.15	0.20	0.18	0.14	0.23	0.15	0.14	0.11
UK	0.24	0.24	0.23	0.18	0.17	0.20	0.16	0.19
Iceland	—	0.22	0.23	0.46	0.24	—	0.00	0.00
Norway	0.22	0.23	0.08	0.19	0.14	0.15	0.11	0.05
Switzerland	0.17	0.17	0.11	0.08	0.07	0.09	0.03	0.07
Serbia	0.09	0.06	0.09	0.14	0.12	0.05	0.05	0.05

As for the determinants of such European differentials (Table 3), the results of our regression model highlight a positive and significant association between SIDS rates and the percentage of females (20–54 years old) with at least upper secondary education. In this case, SIDS mortality increases by 0.4 units for each one‐unit increase in the percentage. The other variables show non‐statistically significant coefficients, although it is possible to appreciate a positive association of SIDS mortality rates with the percentage of foreign births and a concave relationship with the overall IMR rate since we have a negative quadratic coefficient. Indeed, when estimating the model for the entire R group this effect disappears, and the overall model does not result statistically different from the one with only the intercept included (*F* = 1.2; *p* = 0.33).

**Table 3:** Regression analysis of socioeconomic factors on SIDS mortality and R‐group mortality (× 100,000 births). National data from European countries, 2003–2018.

Variables	SIDS		R group	
	Coeff.	*p*‐value	Coeff.	*p*‐value
GDP per capita (in thousand Euros)	−0.002	0.54	0.003	0.62
Pop. 20–54 years with at least upper education (%)	0.006	0.036	0.003	0.44
Foreign births (%)	0.004	0.37	0.001	0.92
Infant mortality rate (× 1000, linear)	0.034	0.61	0.174	0.084
Infant mortality rate (× 1000, quadratic)	−0.004	0.51	−0.017	0.098
Constant	−0.398	0.20	−0.451	0.18
Overall model significance	*F* = 2.9; *p*= 0.038	*F* = 1.2; *p*= 0.33		
Coefficient of determination	*R* ^2^ = 0.304	*R* ^2^ = 0.184		

### 3.2 Individual Analysis

**Figure 4:**
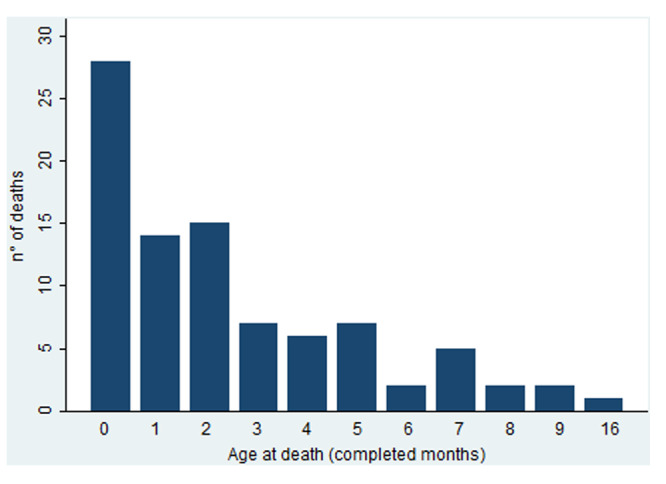
Age at death (in months) of SIDS infants. Individual data, November 2003–November 2018.

The 90 cases forming the individual sample were 47 males and 43 females, values consistent with the sex composition of total infant deaths in Italy (Fisher exact test, *p*= 0.17). As for age at death (in completed months), the modal age is zero months (28 cases, 31.5%), followed by a steady decreasing trend throughout the first year of life. The dataset also includes one very dubious case at the age of 1 year and 4 months (Figure 4).

The share of neonatal deaths from SIDS is significantly lower than the corresponding share among total infant deaths (71.8%), once again confirming the postneonatal nature of this condition. Consistently, the mean age at death is 2.5 ± 2.9 months and the median age is 2.0 months, which is, in this case, to prefer for the presence of the outlier above described, which appears rather exceptional when not suspicious.

No evidence of a significant effect of mother's age at birth on the risk of SIDS was found. Available only for 60 out of 90 cases, infants dead from SIDS had mothers of 30.8 years on average, a value not statistically different from that observed for the general population of Italian mothers in the same period, 31.2 years (*t* = −0.600; *p*‐value = 0.55).

Cold months (winter and autumn) account for 60.7% of total SIDS deaths and although the share is about 50% for total infant deaths, there is no sufficient statistical evidence to highlight a significant difference between the two seasonal distributions (*χ* ^2^ = 4.922, *p*‐value = 0.18).

## 4 Discussion

Italy has one of the lowest values of infant mortality in Europe for a long time [10, 23], but despite the limited number of infant deaths, the impact of SIDS is very limited, with an overall mean annual rate of around 4 deaths per 100,000 births in the period 2003–2018. Low figures of SIDS mortality characterize also the postneonatal period, where, according to the literature [2], they should be far more concentrated, to such a point that some authors consider SIDS deaths in the first month of life so unlikely to exclude them from their analyses, thus assuming an error in the diagnosis of the death cause [20].

The lower rates of SIDS in Italy compared to Europe could be attributed to multiple factors. One possible explanation is the adherence to safe sleep recommendations, including the back to sleep campaign. In Italy, a national campaign on awareness of supine sleep positioning has been widely promoted by perinatal health services. In addition to hospitals, there is also an Italian system of family pediatricians, which is not present in most other European countries. This provides an additional layer of screening to help prevent the risk of SIDS, offering greater health support to families. Cultural factors play a significant role. In Italy, there is a long‐standing tradition of room‐sharing without bed‐sharing, which has been associated with a reduced risk of SIDS. Breastfeeding rates in Italy are also high, particularly during the first years of life, with a protective effect against SIDS. Moreover, differences in data reporting and classification criteria for sudden infant deaths across countries may contribute to the observed variation in SIDS rates. Some cases that might be classified as SIDS in other countries could be recorded under different categories in Italy, such as ‘Sudden unexpected death in infancy’ (SUDI) or other unspecified causes of death, potentially leading to lower reported rates of SIDS.

For what concerns sex differentiation, a larger male disadvantage in SIDS mortality than in infant overall mortality, was observed. This aspect could be influenced by gender differentials in arousal sensitivity after birth [22].

There is evidence [22] that at 2–4 weeks, male infants were easier to arouse than female infants during quiet sleep, while there were no significant effects of gender on total arousability at 2–3 months. According to Richardson et al. [23], although male infants are at greater risk of SIDS than female infants, this difference is unlikely to be associated with gender differences in cortical arousal threshold or frequency.

Along with age and gender differential effects, some literature points also at the role of cultural/ethnic features [24]. Our data do not support that claim, basically for two main reasons. First, in Italy, legal and illegal foreign mothers and their children have completely free access to public hospitals and healthcare institutions. Second, aggregate data lack information on nationality for a non‐marginal fraction of cases, around 14% and 7%, respectively, for overall infant and SIDS mortality.

Such a limited impact of SIDS in Italy places it among the European countries with the lowest figures of SIDS mortality, together with some countries from the Balkans, the Netherlands, and Switzerland. Although SIDS has always been associated with economic hardship, low educational attainment, and peculiar cultural aspects related to the care and growth of infants [24], when looking at the role of some possible determinants of SIDS differentials among European countries, however, none of the factors investigated showed statistical association with national SIDS and R‐group mortality rates. This finding could suggest that national SIDS levels are largely affected by the different ways in which countries record and/or diagnose SIDS deaths. Countries where the system of death certification is less rigorous could count a smaller number of SIDS deaths and have correspondingly a higher number of deaths for unknown or unspecified reasons. At the same time, developed countries whose death certification process is more scientifically rigorous and the consent process for autopsy less restrictive might as well experience low levels of SIDS mortality for their capacity to correctly classify infant deaths.

The individual analysis adds other pieces to the puzzle of SIDS mortality. First, it allows a more refined determination of the age pattern, which in our case study imposes calculation of the median instead of the simple arithmetic means for the suspicious attribution to SIDS of a death of a child of over 1 year of age. Second, it allows to consider social and demographic factors not usually available in aggregate statistics, such as mother's age at birth and season of birth. The mean values of both variables were estimated to be consistent with the mean values of the overall female Italian population, which seems to reinforce the idea of a syndrome scarcely related to demographic factors.

## 5 Conclusions

Despite the epidemiological variability of SIDS in different European countries, Italian SIDS mortality rate is significantly lower than the EU average. SIDS mortality is greater in the postneonatal age than in other critical periods and is also characterized by a higher gender differential to the detriment of the male sex. SIDS mortality is independent of maternal age as well as economical, educational, and cultural aspects related to the care of infants. On the other hand, a wide discussion could be opened on the different ways in which countries record and/or diagnose SIDS deaths, and discrepancies emerge on the way to correctly classify infant deaths.

Data suggests urgent need to get insight into previously unexplored aspects. such as neuroanatomical, genetic, metabolic, and proteomic aspects, focusing especially on SIDS high‐risk groups as models for the etiopathogenesis of this syndrome. At the same time, it is very important for parents to gain awareness about SIDS as recently highlighted through specific awareness measurement tools [25]. In relation to this, the wide involvement of the Italian health system in sensibilization campaigns in collaboration with pediatricians and neonatologists could be the added value responsible for the lower SIDS incidence.

## Author Contributions

**Matteo Manfredini and Serafina Perrone:** investigation, methodology. **Serafina Perrone:** conceptualization, project administration. **Alessia Ardenghi:** methodology, writing – original draft. **Anna Maria Lavezzi:** resources, supervision, writing – original draft. **Virginia Beretta:** writing – original draft, writing – review and editing. **Elena Scarpa:** writing – review and editing. **Sabrina Moretti:** supervision, writing – review and editing. **Susanna Maria Roberta Esposito:** supervision, writing – review and editing. **Laura Filonzi:** investigation, methodology. **Francesco Nonnis Marzano:** supervision, validation.

## Ethics Statement

The data collection was performed in accordance with relevant guidelines and Italian regulations. In particular, clinical data was obtained from the Lino Rossi Center under a permanent ethical permission attributed by the Italian Law n. 31/2006 “Regulations for Diagnostic Post Mortem Investigation in Victims of SIDS and Unexpected Fetal Death” and Decree of the Italian Health Ministry (October 07, 2014). More precisely, the Lino Rossi Research Center is the national reference center for sudden unexplained fetal and infant death. The law decrees that all infants with suspected SIDS who died suddenly in Italian regions within the first year of age, as well as all fetuses who died without any apparent cause, must undergo an in‐depth anatomopathological examination and a complete revision of the case clinical history is permitted. All necroscopic analyses were authorized with an informed consent released by parents or legal guardians in accordance with the Law 31/2006.

## Conflicts of Interest

The authors declare no conflicts of interest.

## Transparency Statement

The lead author Serafina Perrone affirms that this manuscript is an honest, accurate, and transparent account of the study being reported; that no important aspects of the study have been omitted; and that any discrepancies from the study as planned (and, if relevant, registered) have been explained.

## Data Availability

Public Italian and European data analyzed in the current study were obtained from the Italian National Institute of Statistics (ISTAT) and the European statistical office (EUROSTAT). On the other hand, individual‐level datasets are not publicly available because they belong to Lino Rossi Research Center at the University of Milan but are available from the corresponding author on reasonable request.
